# Delayed expression of apoptosis in human lymphoma cells undergoing low-dose taxol-induced mitotic stress

**DOI:** 10.1038/sj.bjc.6600905

**Published:** 2003-05-13

**Authors:** R Allman, R J Errington, P J Smith

**Affiliations:** 1 Cancer Research Wales Laboratories, Velindre NHS Trust, Whitchurch, Cardiff CF14 2TL, Wales, UK; 2 Department of Pathology, University of Wales College of Medicine, Heath Park, Cardiff CF14 4XN, Wales, UK

**Keywords:** paclitaxel, cell cycle, mitosis, apoptosis, p53

## Abstract

The links between low-dose range taxol-induced mitotic arrest and the subsequent engagement of apoptosis are important for identifying the routes to therapeutic action. Here we have investigated the timing of cell-cycle perturbation and cell death responses following continuous exposure to clinically relevant drug concentrations (1–20 nM). Following 8 h of exposure to taxol, the cell line DoHH2 (p53 wild type) exhibited mitotic arrest and engagement of apoptosis, whereas the cell line SU-DHL-4 (p53 mutant) breached cell-cycle arrest with progression to an abnormal cycle and a 24 h delay in the engagement of apoptosis. Imaging showed equivalent dysfunction of mitotic spindles in both cell lines. The results of kinetic analyses indicated that although cell death may occur at different stages of progression through mitosis and subsequent cell cycles, the overall kinetics of cell death relate to the rate of arrival at a critical event window in the cell cycle. We propose a simple model of low-dose taxol-induced cell death for cycling populations in which mitotic stress acts as a primary trigger for apoptosis with equivalent but potentially delayed outcomes. This view provides a rationale for the clinical effectiveness of this agent, independent of the initial capacity of the tumour cell to engage apoptosis due, for example, to mutant p53 expression. The results provide a perspective for the design of combination regimens that include low-dose taxol and a component that may disturb mitotic delivery.

Paclitaxel (taxol) is a complex diterpenoid isolated from the inner bark of the Western yew tree – *Taxus brevifolia* (Wani *et al*, 1971). This naturally occurring agent has been shown to have significant activity against advanced ovarian cancers (McGuire *et al*, 1989; Runowicz *et al*, 1995), metastatic breast cancers (Holmes *et al*, 1991; Hortobagyi and Holmes, 1996), and leukaemias (Rowinsky *et al*, 1989) refractory to standard chemotherapy. It has also shown some activity against various other tumour types (Etlinger, 1993; Forastiere, 1993; Einzig and Wiernik, 1995; Edelman and Gandara, 1996). Although the mechanism by which stable microtubules are induced by taxol are well established (Schiff *et al*, 1979; Rao *et al*, 1992, 1994), the molecular links between tubulin polymerisation, cell-cycle arrest, and ultimately cell death are not so well characterised. Given the broad spectrum of activity of taxol with regard to tumour type and genetic background, it is important that these mechanisms are resolved. In particular, whether a mitotic arrest is essential for taxol-induced cell death and if so does a functional spindle checkpoint affect survival outcome. Many successful cancer treatments rely upon combinations of therapeutic agents, and it is important for the development of such regimens so that the mechanism and the complex kinetics of cellular response to taxol are clarified. In particular, combination with agents that have different cell-cycle specificities may produce effects that abrogate taxol action.

Taxol appears to target microtubules specifically both *in vitro* and *in vivo* (Schiff and Horwitz, 1980, 1981; Manfredi *et al*, 1982; Manfredi and Horwitz, 1984; De Brabender *et al*, 1996). However, unlike classical antimicrotubule agents, such as colchicine and vinblastine, that induce microtubule disassembly and paracrystal formation (Wilson *et al*, 1974), the antitumour activity of taxol has been demonstrated to be associated with microtubule stabilisation (Manfredi and Horwitz, 1984; Horwitz *et al*, 1986) and with the arrest of cells in the G2/M phase of the cell cycle (Rowinsky *et al*, 1990; Slichenmyer and von Hoff, 1991; Rowinsky *et al*, 1992). In addition to stabilising microtubules and inducing cell-cycle arrest, taxol has also been demonstrated to induce apoptosis in a variety of solid tumour cells (Cheng *et al*, 1995; Jordan *et al*, 1996; Milross *et al*, 1996). However, the dependence of taxol-induced cell death on cell-cycle-specific arrest is not clear. Apoptosis is an important factor in describing population responses to DNA damage and is related in part to p53 function. For example, while wild-type p53 cells undergo apoptosis after exposure to radiation, p53 mutant cells show delays in the progression to apoptosis (Lowe *et al*, 1993b). Dysfunction of p53-dependent pathways can allow tumour cells to evade the checkpoint controls and apoptosis, thereby gaining a selective advantage in attempting to overcome therapy (Lowe *et al*, 1993a). As a consequence, cancers with TP53 mutations tend to have a worse prognosis for responses to chemotherapy than those showing wild-type alleles (Callahan, 1992).

The situation regarding the role of TP53 dysfunction in the response of tumour cells to taxol is more complicated with several contradictory reports appearing in the literature. One report describes that in different cultured fibroblast cell lines, disruption of wild-type p53 function resulted in increased sensitivity of cells to taxol treatment compared to cells expressing wild-type p53 (Wahl *et al*, 1996). Conversely in an ovarian cancer cell line expressing wild-type p53, the disruption of p53 by transfection with the E6 protein of human papilloma virus type 16 led to a decrease in sensitivity to taxol (Wu and El-Deiry, 1996). It has also been reported that the introduction of wild-type p53 into an ovarian cancer cell line not expressing p53 resulted in no change to the tumour cell sensitivity to taxol (Graniela Sire *et al*, 1995). The situation is further complicated with taxol being reported to increase the levels of p53 protein in some cell types (Tishler *et al*, 1995) but not in others (Graniela Sire *et al*, 1995).

Regardless of p53 status, the sequence of signals triggering the apoptotic response may also be dependent upon taxol concentration. Thus, when cells are exposed to low concentrations of taxol (1–100 nM), which do not affect the overall cytoskeletal microtubule structures (Jordan *et al*, 1993), it has been proposed that triggering of the mitotic spindle checkpoint promotes arrest and apoptosis directly (Sorger *et al*, 1997). The mitotic spindle checkpoint is a signal transduction pathway that links a kinetochore-associated sensor, which monitors the appropriate connection between mitotic spindles and chromosomes, to an output that arrests the cell cycle (Sorger *et al*, 1997). Several mammalian gene products have been identified to function at the spindle assembly checkpoint including the Mad (mitotic arrest defective) proteins Mad 1-3, the Bub (budding uninhibited by benomyl) proteins, Bub 1-3 and Mps1. Treatment with taxol or nocodozole upregulates Bub3 (Martinez-Exposito *et al*, 1999) and expression of dominant-negative Bub1 inhibits nocadazole-induced apoptosis (Taylor and McKeon, 1997), suggesting that protracted activation of these gene products may play a role in apoptosis induction following a mitotic arrest. It has also been proposed that abnormal mitotic exit provides the signal for apoptosis (Jordan *et al*, 1996). Wang *et al* (2000), suggesting that these two events are the two possible triggers for cell death following mitotic arrest or delay. Our hypothesis was that arrival of cells at this critical cell-cycle phase is the ‘event’ that triggers processes that end in cell death, although timing of the overt expression of cell death will depend upon genetic status. Thus, the rate of delivery to that event window determines future expression of cell death, and the cell-cycle position at which cell death actually occurs may be misleading in terms of event linkage.

This study was designed to identify the links between mitotic arrest, and subsequent engagement of apoptosis, by investigating the timing of these responses following exposure to clinically relevant concentrations of taxol and over a sufficiently long period to identify fully response outcomes. This is important since different routes of taxol evasion prior to commitment to cell death could provide routes for drug resistance phenotype development. We have used two cell lines that represent different extremes of the apoptotic responses to taxol in a multiparameter flow cytometric study, which delineated cell-cycle traverse and correlated landmark cell-cycle events with the subsequent engagement and kinetics of apoptosis. Cell-cycle landmarks were confirmed using dual parameter measurements of DNA and either cyclin B1 (Gong *et al*, 1995; Widrow *et al*, 1997) or the mitosis-specific protein MPM2 (Juan *et al*, 2001).

## MATERIALS AND METHODS

### Cell lines and culture conditions

Two human follicular B-lymphoma cell lines were used in this study. DoHH2 was a kind gift from Dr JC Kluin-Nelemans [Leiden, The Netherlands; (Kluin-Nelemans *et al*, 1991)], and SU-DHL-4 was provided by Professor FE Cotter (Epstein *et al*, 1978). DoHH2 was routinely maintained in RPMI 1640 supplemented with 5% FCS. SU-DHL-4 was cultured in RPMI 1640 supplemented with 10% FCS. All growth media were supplemented with 100 U ml^−1^ penicillin, 100 *μ*g ml^−1^ streptomycin, and 2 mM glutamine. The cells were passaged twice weekly at an initiating density of 5 × 10^4^ cells ml^−1^ cultured at 37°C in a humidified atmosphere of 5% CO_2_/95% air. The p53 status of DoHH2 (wild-type radiation response) and SU-DHL-4 (stabilised mutant) was confirmed by immunoblotting.

### Irradiation

All irradiations were performed at room temperature in oxic conditions using a ^137^Cs gamma source of 0.66 MeV energy. The dose rate was 1.2 Gy min^−1^. Irradiations were usually completed within 10 min.

### Flow cytometry

Flow cytometric analyses were performed using a standard configuration FACScan equipped with an argon-ion laser (Becton Dickinson, Oxford, UK). Data from either 6000 or 10 000 cells were recorded using CellQuest software (Becton Dickinson, Oxford, UK). Data analysis was performed using WinMDI (J Trotter, Scripps Research Institute, San Diego, USA). Cell debris was excluded from all the measurements on the basis of forward and orthogonal light scatter. In the case of DNA analysis, cell aggregates were excluded on the basis of pulse-width *vs* pulse-area measurements of propidium iodide fluorescence. Normal human lymphocytes were used to monitor instrument performance and calibrate measurements of cellular DNA content.

### Single parameter DNA measurements

Cell cultures were resuspended in phosphate-buffered saline (PBS). A volume of 125 *μ*l of propidium iodide (0.4 mg ml^−1^)/Triton X-100 (1% v v^−1^) was added to 1 ml of cell suspension together with 50 *μ*l Ribonuclease A (10 mg ml^−1^) (Sigma, Poole, UK). Cells were incubated at 37°C for 10 min prior to FACS analysis. Normal human lymphocytes were used to monitor instrument performance and to calibrate measurements of DNA ploidy. Where required, DNA histograms were deconvoluted according to the protocol of Watson *et al* (1987) and Ormerod *et al* (1987).

### Annexin-V–FITC measurements

In total, 0.5 ml cells (5 × 10^5^ cells) were labelled using an Annexin-V–FITC labelling kit (Calbiochem-Novabiochem, Nottingham, UK). Briefly, cells were labelled directly in the culture medium using 1.25 *μ*l Annexin-V–FITC and 10 *μ*l of the supplied media binding reagent for 15 min at room temperature in the dark. Cells were centrifuged at 250 **g** for 5 min and resuspended in 0.5 ml of binding buffer (10 mM HEPES pH 7.4, 150 mM NaCl, 2.5 mM CaCl_2_, 1 mM MgCl_2_, 4% BSA). A volume of 10 *μ*l propidium iodide (30 *μ*g ml^−1^) was added and the samples analysed immediately by flow cytometry.

### Dual parameter cyclin B1/DNA and mitotic proteins/DNA measurements

Approximately 5 × 10^5^ cells were collected for each sample. Cells were washed with cold PBS, fixed in ice-cold 70% ethanol, and then stored overnight at 4°C. Cells were washed in PBS and resuspended in 100 *μ*l PBS containing 1% bovine serum albumin (BSA). A volume of 10 *μ*l anti-cyclin B1 mouse monoclonal antibody (Dako, Ely, UK) or 10 *μ*l anti-mitotic proteins (clone MPM2) mouse monoclonal antibody (Dako, Ely, UK) was added and the cells stored overnight at 4°C. Cells were washed twice in PBS and resuspended in 100 *μ*l PBS containing 1% BSA. A volume of 10 *μ*l anti-mouse IgG–FITC monoclonal antibody (Dako, Ely, UK) was added and the cells incubated for 1 h at room temperature in the dark. Cells were washed twice in PBS to remove excess antibody and resuspended in 1 ml propidium iodide (10 *μ*g ml^−1^). A volume of 50 *μ*l of RNAse (10 mg ml^−1^) was added and the cells incubated for 30 min at room temperature prior to FACS analysis.

### Microtubules immunocytochemistry

Approximately 10^6^ cells were collected for each sample. Cells were washed with cold PBS, fixed in 4% paraformaldehyde, and then stored overnight at 4°C. Cells were washed in PBS and rehydrated in PBS for 10 min at room temperature. Cells were permeabilised in 0.1% Triton X-100/PBS for 30 min at room temperature. Nonspecific antibody labelling was blocked using goat serum (1 : 10 dilution) for 1 h at room temperature. Anti-*β*-tubulin monoclonal antibody (clone TUB 2.1) (Sigma, UK) was added (1 : 200 dilution) for 1 h at 37°C. Cells were washed four times to remove excess antibody. Goat-anti-mouse IgG-Cy3 monoclonal antibody (Amersham Pharmacia, Cardiff, UK) was added (1 : 100 dilution) for 30 min at 37°C. Cells were washed three times in PBS to remove excess antibody. DNA was labelled using the red fluorescent probe DRAQ5 (Biostatus Ltd, Shepshed, UK) (Smith *et al*, 2000a). Cells were loaded with 10 *μ*M DRAQ5 for 10 min at room temperature and mounted without further washing.

### Confocal laser scanning microscopy

A single optical slice dual channel image was acquired using a confocal laser scanning microscope (CLSM) (BioRad Microsciences Ltd, Hemel Hempsted, UK), equipped with a krypton/argon ion laser and attached to a Zeiss Axiovert 135. DRAQ5 (DNA) was visualised using 647 nm excitation and detected at 680/30 nm. Cy3 (tubulin) was excited at 568 nm and detected at 590/40 nm.

## RESULTS

### DNA damage response and cell-cycle checkpoint status

**Figure 1:**
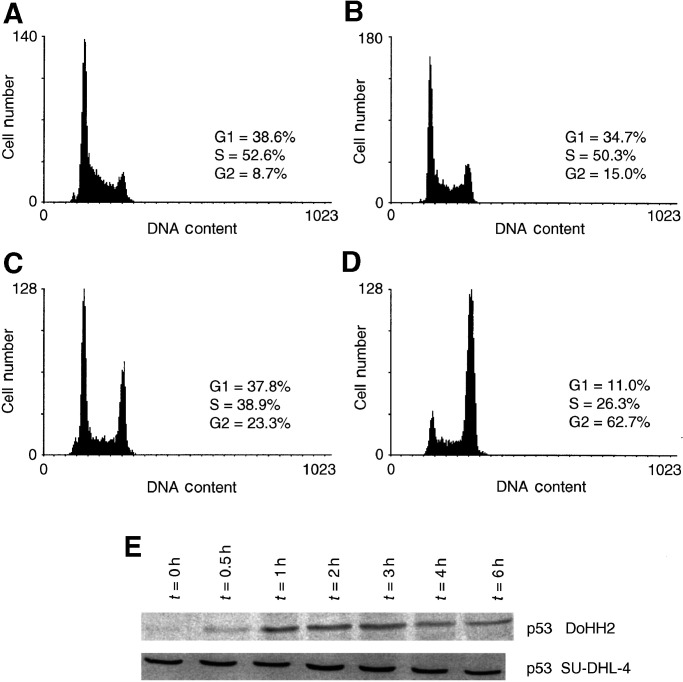
Differential cell-cycle arrest in response to ionising radiation. DNA distributions are (**A**) DoHH2 and (**B**) SU-DHL-4 under normal exponential growth conditions, and obtained 14 h following exposure to 4 Gy of ionising radiation (**C**) DoHH2, (**D**) SU-DHL-4. Data are representative distributions obtained from 6000 cells. (**E**) SDS–PAGE followed by immunoblotting for p53, analysis of cellular lysates from the two cell lines prepared at the indicated time points after irradiation at 6 Gy.

The two cell lines SU-DHL-4 and DoHH2 both grow in suspension culture with doubling times of approximately 17 and 21 h, respectively. To establish whether the two cell lines exhibit the expected differential pattern of cell-cycle arrest in response to ionising radiation (based upon their known p53 status), cultures were exposed to 4 Gy of *γ*-irradiation (preliminary observations indicated that this dose was suitable for inducing cell-cycle arrest without inducing a substantial cell kill). Figure 1

shows the typical changes in cell-cycle distribution of irradiated DoHH2 and SU-DHL-4 cells with a postirradiation incubation period of 14 h. The DoHH2 cell line exhibits a retention of cells in G1 and an accumulation in G2 with a simultaneous reduction in the number of cells in S phase, typical of a wild-type p53 response (Kuerbitz *et al*, 1992). In contrast, the SU-DHL-4 cells lack a G1/S arrest phenotype in response to ionising radiation and exhibit a greater accumulation of cells in G2/M with a concomitant reduction of the number of cells in both G1 and S phase, typical of a mutated p53 response (Kuerbitz *et al*, 1992). SU-DHL-4 cells were also more resistant to radiation-induced apoptosis than the DoHH2 cell line (data not shown). Immunoblotting confirmed the rapid radiation-induced changes in stabilisation of p53 in DoHH2 but not in SU-DHL-4.

### Taxol induces a G2/M arrest in both DoHH2 and SU-DHL-4

**Figure 2:**
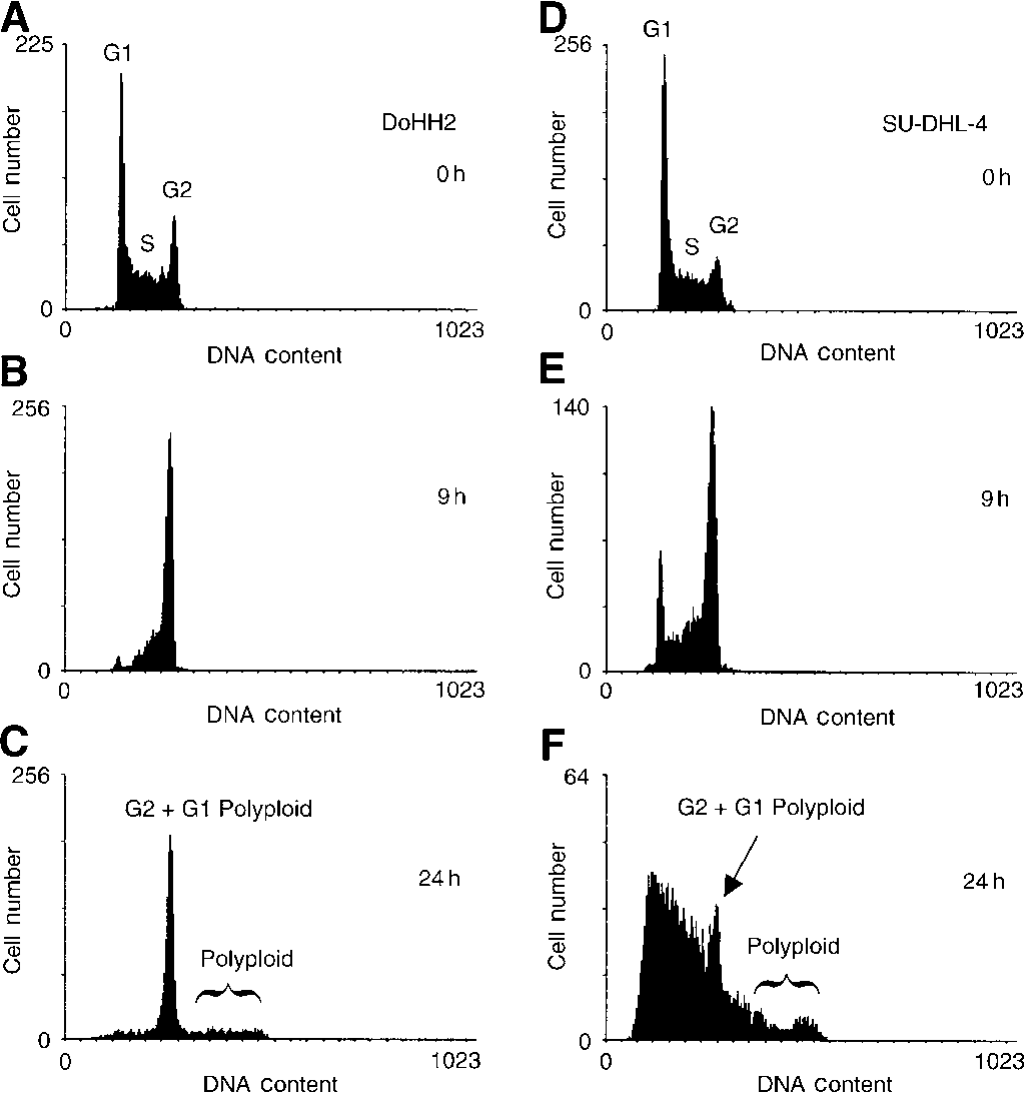
Differential G2/M arrest and progression to polyploidy in DoHH2 and SU-DHL-4 following continuous exposure to taxol. Cells were incubated with 10 nM taxol for the indicated times.

To establish the general characteristics of the cell-cycle response to taxol, cells were exposed to taxol at varying concentrations in the clinically relevant low-dose range 1–20 nM (Huizing *et al*, 1993). The preparation method for cell-cycle analysis does not require cell washing and, therefore, provides a high degree of retention of both apoptotic and mitotic nuclei (Smith *et al*, 2000b). Figure 2

shows typical DNA histograms obtained from both cell lines following treatment with 10 nM taxol. At 9 h following addition of taxol both cell lines exhibit an accumulation of cells in G2/M, this accumulation being more pronounced in the DoHH2 cell line, with 43% of the cells being in G2/M as opposed to 30% of the SU-DHL-4 line. Following 24 h of exposure to taxol, the majority of the DoHH2 cells (58%) remain in a cell-cycle arrest. In the SU-DHL-4 line however, only 10% of cells were retained in G2/M with the majority of cells appearing to have progressed to a polyploid generation demonstrating aberrant DNA contents. Thus, it appears that both cell lines are responding to taxol by showing compromised traverse of G2/M with cell-cycle arrest evident in DoHH2, whereas the duration of any arrest appears to be greatly reduced in the SU-DHL-4 (*TP53-*mutant) cell line.

**Figure 3:**
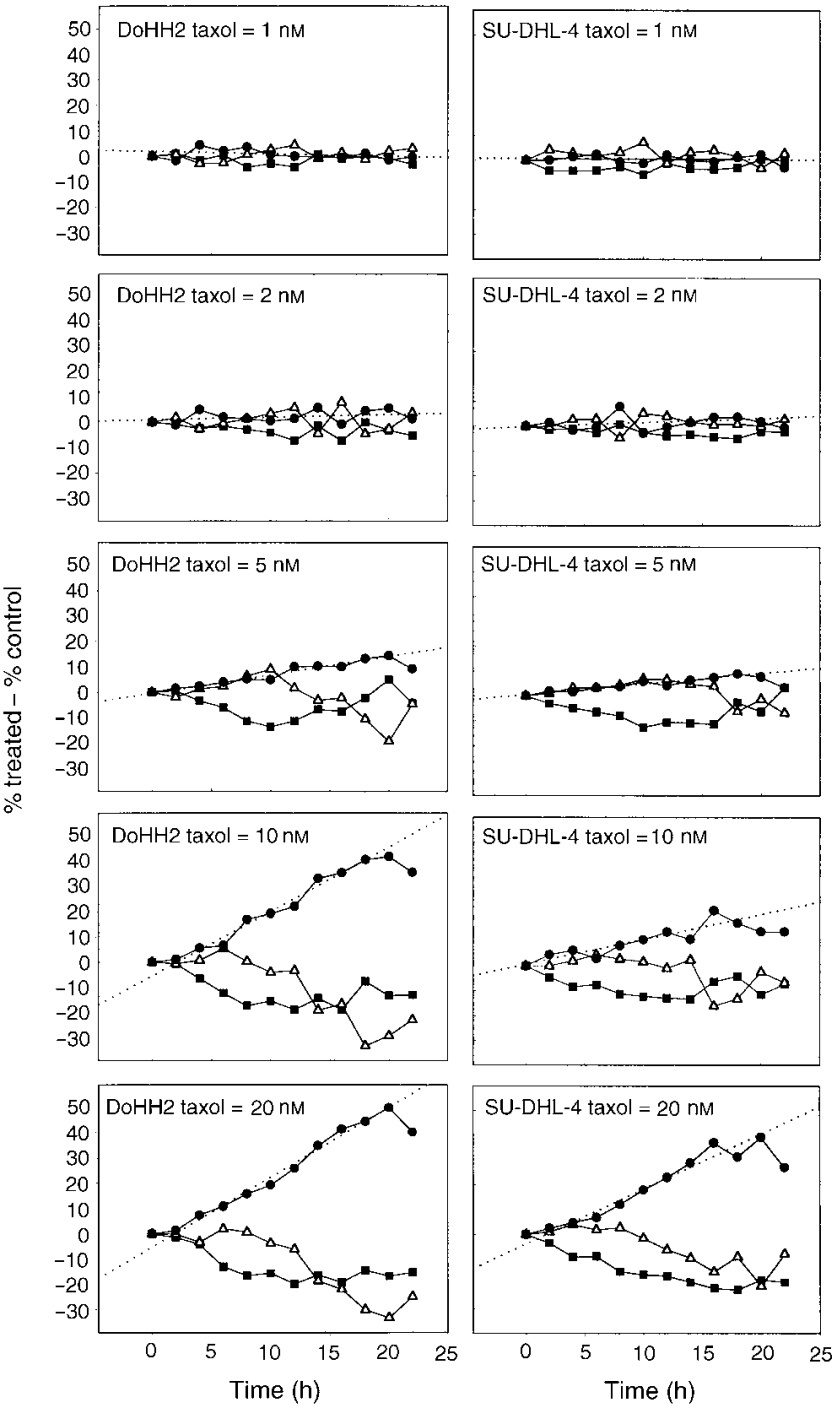
Dose dependency of the kinetics of cell-cycle perturbation induced in DoHH2 and SU-DHL-4 by continuous exposure to taxol at 2, 5, 10, or 20 nM. DNA histograms were deconvoluted and the results plotted as percentage of cells in each particular cell-cycle phase, G1 (▪), S (▵), G2/M+G1 polyploid (•), relative to those of an untreated population. The dotted line in each panel represents a linear regression analysis of the G2/M curve. The rates (%/h) of accumulation in G2/M following exposure to 1, 2, 5, 10, or 20 nM taxol are: DoHH2=0.09, 0.11, 0.72, 2.55, 2.76; SU-DHL-4=0.02, 0.17, 0.43, 1.01, 2.22.

Deconvolution of DNA content distributions obtained from cultures exposed to varying concentrations of taxol over a 24 h time period permitted the plotting of the kinetics of cell-cycle perturbation induced by taxol (Figure 3

). Both DoHH2 and SU-DHL-4 respond to taxol by a dose-dependent arrest in G2/M, with a concomitant reduction of the fraction of cells in both the G1 and S phase. The results indicate that taxol elicits a cell-cycle arrest in DoHH2 cells at concentrations of 5, 10, and 20 nM, and that this arrest is substantially maintained during the period of exposure to the drug. Although the same concentrations of taxol induce a cell-cycle arrest in SU-DHL-4, the magnitude of this arrest is reduced compared to that for the DoHH2 line. Linear regression analysis of the data indicated that DoHH2 and SU-DHL-4 both exhibited a near maximal rate of accumulation in G2/M following exposure to 20 nM taxol, with rates of accumulation being 2.8 and 2.2% per h, respectively. These rates of accumulation were maintained for approximately 20 h, that is, approximately one full cell-cycle traverse period. However, an analysis of the DNA distributions (see Figure 2) indicates that at later time points (>10 h), both cell lines show evidence of progression to polyploidy, therefore the data plotted as G2/M will also contain G1 polyploid cells.

**Figure 10:**
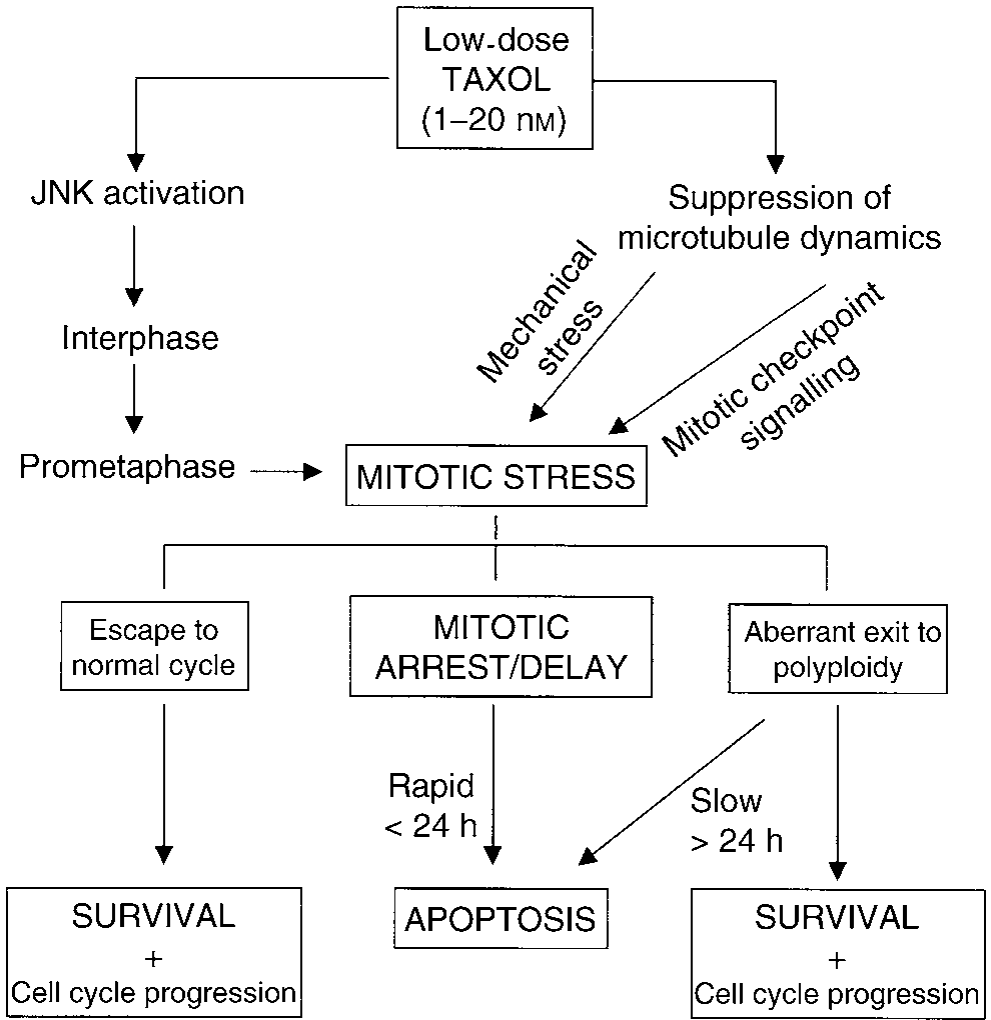
Proposed model of taxol-induced cell death (incorporating concepts from Wang *et al*, 2000) in which cells may either undergo a rapid engagement of apoptosis during mitotic arrest or a delayed engagement of apoptosis following aberrant mitotic exit.

Thus, following taxol-induced cell-cycle arrest there are three possible outcomes for the cell depending on the dose of drug to which they are exposed. At low doses, cells may escape cell-cycle arrest and continue cycling normally. At higher doses, cells may experience a sustained cell-cycle arrest, or alternatively cells may breach the cell-cycle arrest and proceed to polyploidy. These three possible cell-cycle arrest events provide the key decision points that determine whether a cell subsequently survives the cytotoxic insult by taxol or proceeds to a programmed cell death (see Figure 10

).

### Taxol induces a sustained cell-cycle arrest in DoHH2 cells and a transient arrest in SU-DHL-4 cells

**Figure 4:**
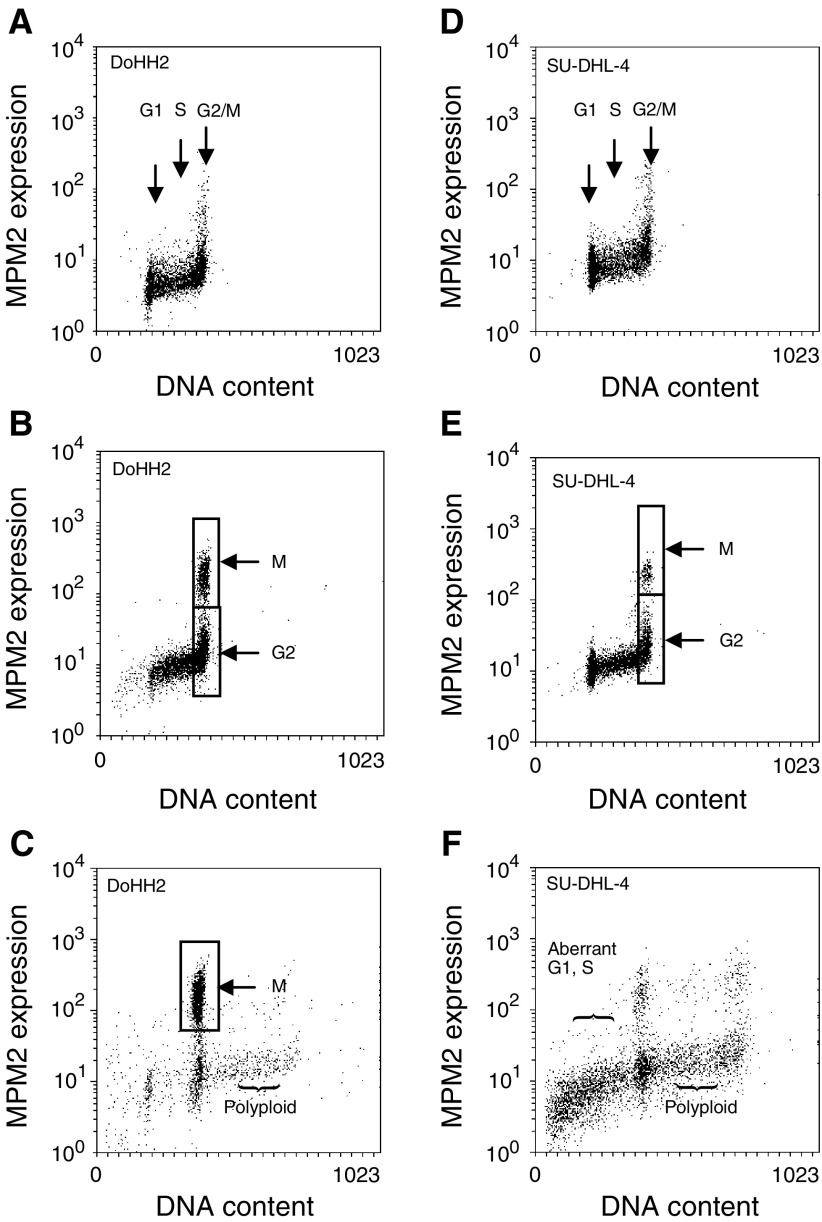
Dual parameter measurement of the mitotic protein MPM2 expression *vs* DNA content obtained during normal exponential growth (**A**) DoHH2, (**D**) SU-DHL-4, and following 8 h exposure to 10 nM taxol (**B**) DoHH2, (**E**) SU-DHL-4, and following 24 h exposure to 10 nM taxol (**C**) DoHH2, (**F**) SU-DHL-4. Data are representative distributions obtained from 10 000 cells.

The differential pattern of cell-cycle arrest between the two cell lines was confirmed by dual parameter measurements of the mitosis-specific protein MPM2 *vs* DNA content (Figure 4

). It can be seen that following an 8 h exposure to 10 nM taxol, both cell lines show evidence of a subpopulation of MPM2-positive cells. However, this subpopulation is reduced in the SU-DHL-4 line as compared to the DoHH2 line. It is clear that following 24 h exposure to 10 nM taxol, the majority of the DoHH2 cells remain arrested in mitosis, while the majority of the SU-DHL-4 cells have shown cycle progression and exhibit aberrant and polyploid DNA contents, which was also demonstrated in the single parameter DNA measurements (see Figure 2).

**Figure 5:**
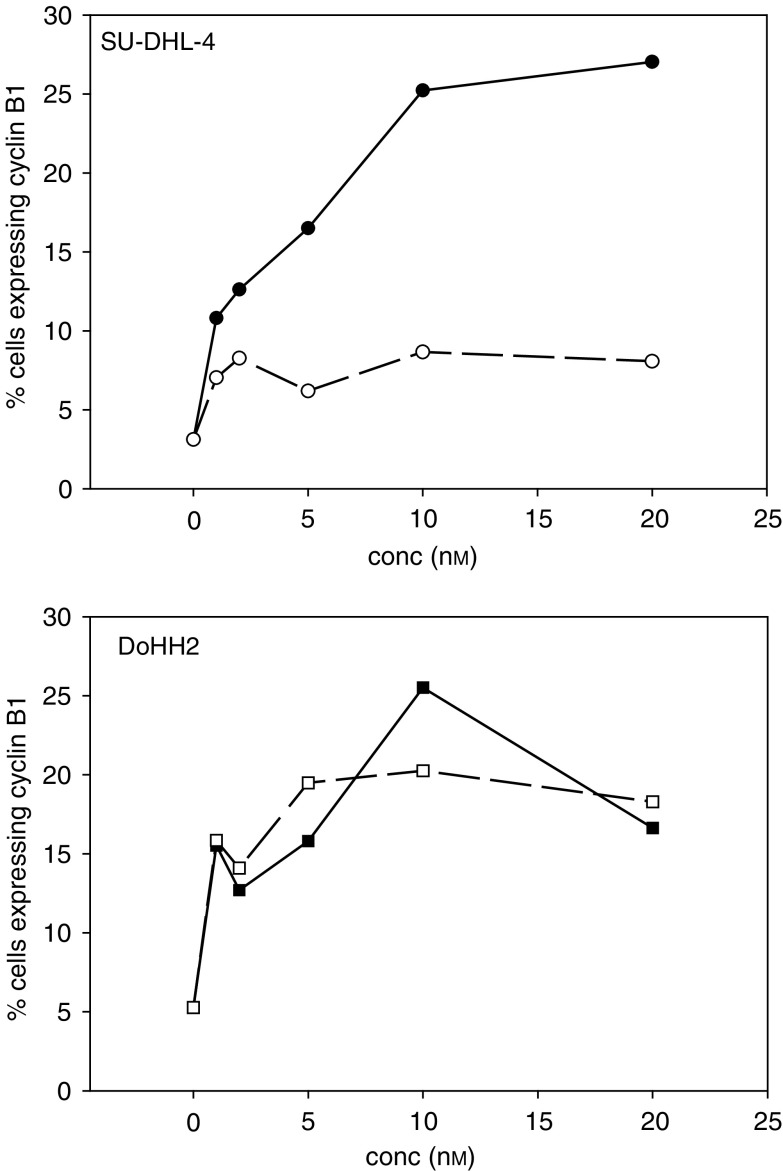
Changes in the percentage of cells expressing cyclin B1 (**A**) SU-DHL-4 and (**B**) DoHH2 following either 8 h (closed symbols) or 24 h (open symbols) of exposure to 0, 1, 2, 5, 10, 20 nM taxol. Cells were labelled with anticyclin B1 monoclonal antibody and analysed by flow cytometry. Data shown from a representative analysis of 6000 cells.

To confirm the observed differences in the duration of mitotic arrest, we also measured cyclin B1 protein expression following exposure to taxol. Cyclin B1 is primarily synthesised during G2 and is rapidly degraded at the end of mitosis (Pines and Hunter, 1989). Dual parameter flow cytometric measurements of cyclin B1 expression *vs* DNA content following 8 h of exposure to taxol consistently indicated that the percentage of cells expressing cyclin B1 during G2/M was increased following exposure to all of the concentrations of taxol tested (1, 2, 5, 10, 20 nM) (Figure 5

). Following a 24 h exposure to taxol however, the number of SU-DHL-4 cells in G2/M expressing cyclin B1 was reduced, at all drug concentrations. In contrast, levels in the DoHH2 line remained similar to those obtained at 8 h. This pattern of results is consistent with the view that the SU-DHL-4 cells are escaping cell-cycle arrest at the 24 h measurement point. However, it is also apparent that both cell lines exhibit increased cyclin B1 expression at doses of taxol that did not induce a measurable G2/M arrest on the basis of single parameter DNA measurements. Cyclin B1 is known to be associated with microtubules (Jackman *et al*, 1995) and our observations suggest that taxol-induced tubulin polymerisation, at concentrations insufficient to induce a cell-cycle arrest, can stabilise cyclin B1 levels.

### Taxol induces aberrant mitoses in both DoHH2 and SU-DHL-4 cells

**Figure 6:**
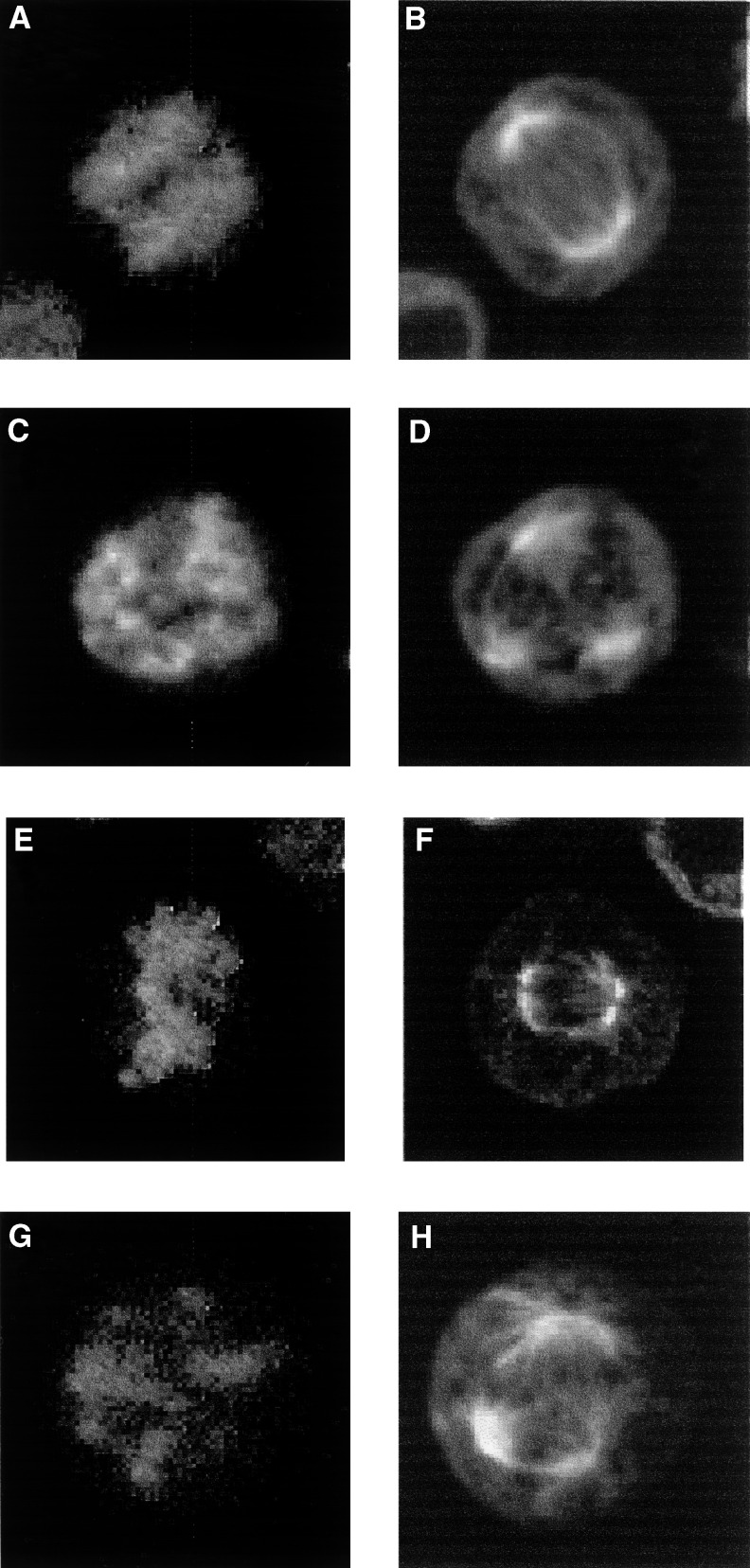
Laser scanning confocal fluorescence images showing normal chromatin segregation and mitotic spindle formation in DoHH2 (**A** and **B**) and SU-DL-4 (**C** and **D**) cells and the aberrant chromosome distribution and tripolar mitotic spindle formation induced by exposure to 20 nM taxol in DoHH2 (**E** and **F**) and SU-DHL-4 (**G** and **H**).

Confocal laser scanning microscopy was performed in order to obtain high-resolution spatial information on tubulin structures and associated chromatin condensation. The results confirmed that taxol induced the formation of aberrant mitotic spindles with a concomitant abnormal chromosome segregation in both cell lines (Figure 6

). All of the observed mitoses in DoHH2 or SU-DHL-4 following exposure to taxol displayed abnormal mitotic spindles and chromatin segregation. These abnormal mitoses were all first cycle events and were similar in appearance for both the cell lines, with the formation of a tripolar mitotic spindle being the predominant aberrant feature following exposure to taxol. In order to confirm the lack of p53-dependency in these observations, we have extended our studies to include observations of isogenic thyroid-derived (K1E7) cell lines, which differ only in TP53 status (Wyllie *et al*, 1999). Using a time-lapse microscopy approach, we have confirmed that taxol induces similar first cycle abnormal mitoses in both wild-type and mutant p53 backgrounds (data not shown).

### Taxol induces cell-cycle arrest in both G2 and mitosis

**Figure 7:**
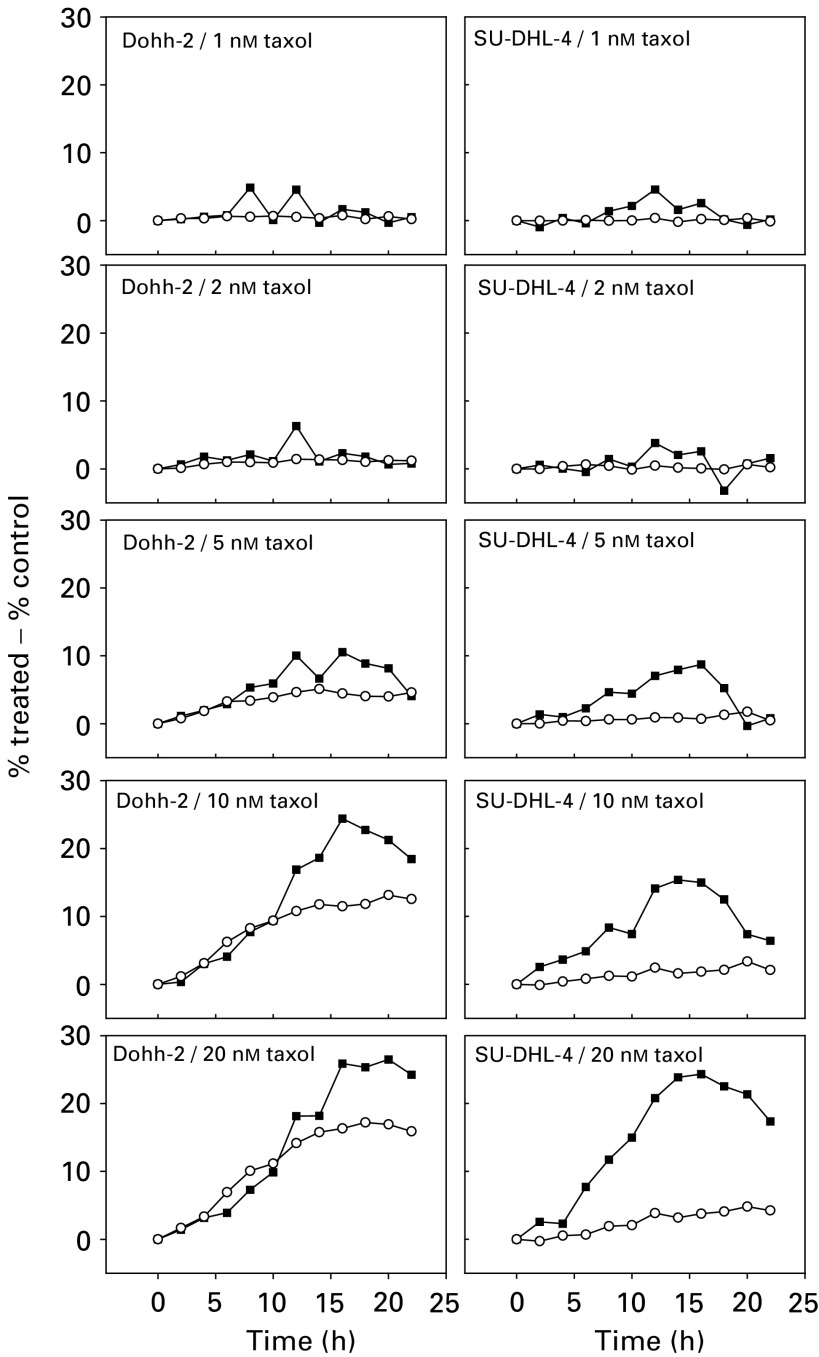
Differential kinetics of cell-cycle arrest in G2 (+G1 polyploid) (○) and mitosis (▪) following exposure to taxol at 2, 5, 10, or 20 nM. Discrimination of G2/G1pol and M was on the basis of side-scatter *vs* DNA content.

Discrimination between G2 and M on the basis of dual parameter side-scatter *vs* DNA content (Epstein *et al*, 1988) allowed us to further clarify the taxol-induced accumulation of cells in G2/M. This methodology detects a mitotic subpopulation that excludes prophase cells and is based on light scatter changes associated with nuclear chromosome condensation and the breakdown of the nuclear membrane that defines the start of prometaphase and continues through until telophase. Figure 7

illustrates the differential accumulation of cells in G2 and mitosis based on this discrimination, following exposure to taxol. It can be seen that taxol induces an accumulation of cells in both G2 and M in both the cell lines. However, in the SU-DHL-4 cell line, the number of cells arrested during mitosis is greatly reduced compared to the DoHH2 line, in agreement with the data presented in Figure 4 and Figure 5. There is also an early and sustained increase in the number of cells with a G2 DNA content. Analysis of single parameter DNA histograms infers that SU-DHL-4 cells do not maintain a mitotic arrest, but proceed to polyploidy. We therefore suggest that the observed increase in the number of cells exhibiting a G2 DNA content is composed both of cells experiencing a G2 delay and also those cells that have undergone an abnormal mitotic exit and are G1 polyploid (with 4N DNA content).

### Taxol induces apoptosis in p53 wild-type and p53 mutant lymphoma cells

**Figure 8:**
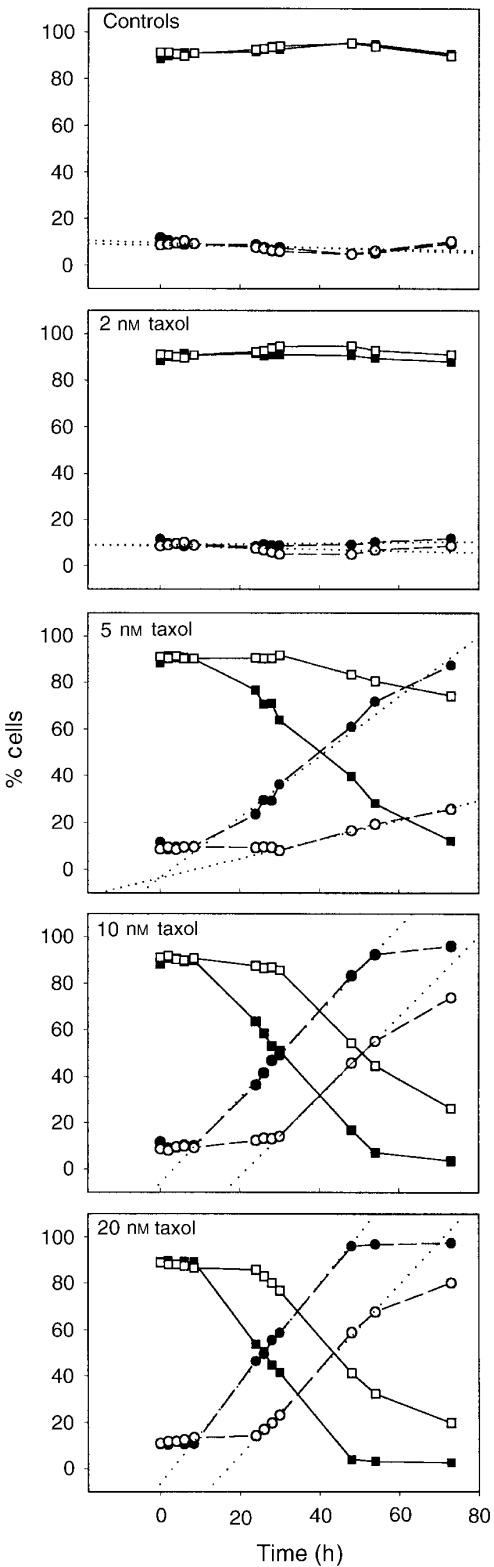
Differential kinetics of the apoptotic response in DoHH2 and SU-DHL-4 following continuous exposure to 0, 2, 5, 10, or 20 nM taxol. Key: Live DoHH2 (▪), annexin-positive DoHH2 (•), live SU-DHL-4 (□), annexin-positive SU-DHL-4 (○). The dotted lines in each panel represent a linear regression analysis of the straight line region of the annexin-positive curves. The rates of appearance of apoptotic cells following exposure to 0, 2, 5, 10, and 20 nM taxol are as follows: DoHH2=−0.05, 0.02, 1.29, 1.84, 2.16; SU-DHL-4=−0.02, −0.03, 0.41, 1.71, 1.84.

Annexin-V–FITC labelling of DoHH2 and SU-DHL-4 cells indicated that both cell lines respond to taxol in a dose-dependent manner by proceeding to apoptosis (Figure 8

); however, the response differs between the two cell lines. Thus, 5 nM taxol elicits over a 90% induction of apoptosis in the DoHH2 cell line during the course of 72 h exposure, whereas only approximately 20% of the SU-DHL-4 line are induced to undergo apoptosis by 5 nM taxol over the same time period. Using these terms of reference, we can state that the DoHH2 cell line is more ‘sensitive’ to taxol than the SU-DHL-4 line.

One striking feature of the data is that it appears that the time for onset of apoptosis for a given cell line (8 h DoHH2; 24 h SU-DHL-4) following drug exposure is remarkably constant and dose-independent, once an apoptotic threshold condition is reached. We can interpret this observation as either a threshold continuous exposure period that is required before cell death occurs, or a fixed delay period (lag) that occurs after the initial insult during which the apoptotic mechanism is engaged. It is apparent from an examination of the data obtained following exposure to 5 nM taxol that a prior cell-cycle arrest *per se* is not a prerequisite for the induction of apoptosis by this agent.

**Figure 9:**
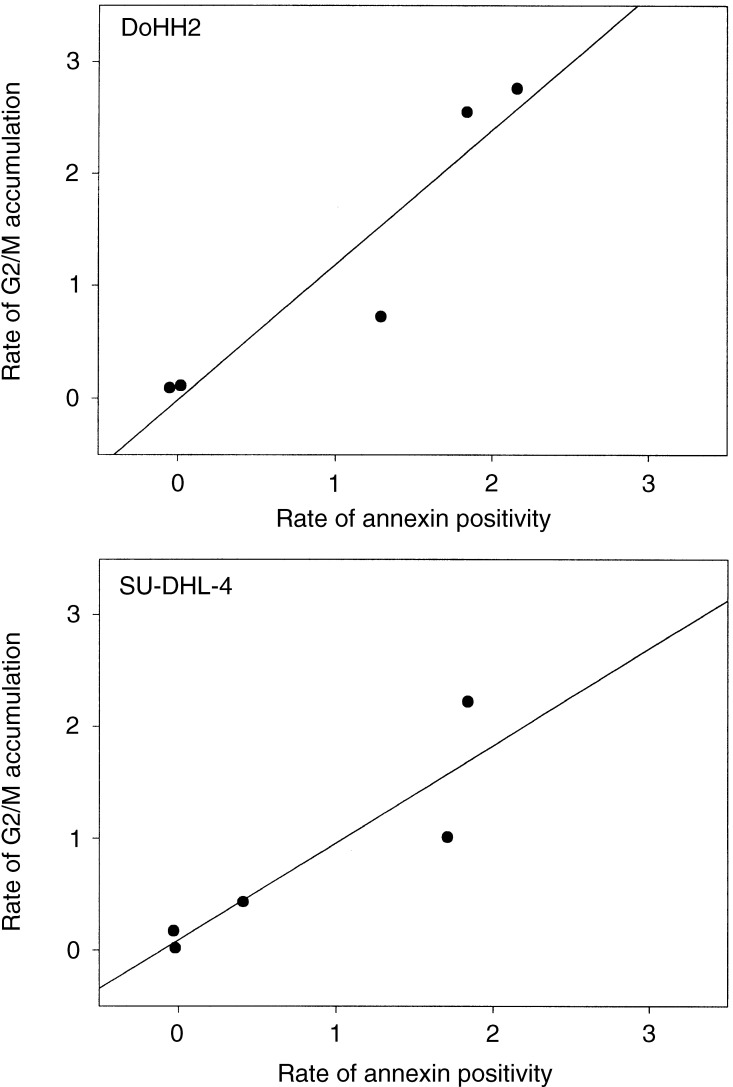
Correlation analysis illustrating the relation between the rate of appearance of annexin-positive cells and the rate of cell-cycle arrest in G2/M for DoHH2 and SU-DHL-4.

Following the initial delay period, once the apoptotic events start to appear, the rate at which the apoptotic population expands with time is remarkably similar for both cell lines and this rate appears to reach a maximum of approximately 2% per h. For a *P*-value <0.05, the correlation coefficients, *r*, for the rate of G2/M accumulation *vs* the rate of appearance of apoptotic cells (annexin positive) are 0.90 for DoHH2 and 0.93 for SU-DHL-4 (Figure 9

). This significant correlation suggests a causal link between the rate at which cells arrive at mitosis (that may or may not comprise a functional checkpoint) and the subsequent rate of apoptosis. Although we have fitted a straight line for both data sets (because of the number of data points), we recognise the possibility of more complex relations between G2/M arrest and apoptosis. We suggest that at low doses (1–5 nM), although cells experience ‘mitotic stress’, it is either insufficient to induce a complete cell-cycle arrest or that many cells rapidly breach or exit this arrest. At higher doses (20 nM), both the rate of G2/M accumulation and apoptosis may be reaching maximum levels. With these considerations aside, the relation between the two events still holds, that is, the prior rate of experience of a cell-cycle perturbation (experience of mitosis) is influencing the subsequent rate of cell death in both the cell types.

## DISCUSSION

This study has provided a detailed time analysis of cell-cycle disruption following continuous exposure to low concentrations (1–20 nM) of taxol. Cell-cycle events have been correlated with the onset and subsequent kinetics of cell death in two lymphoma cell lines that differ in their capacity to execute rapidly apoptosis. Importantly, the concentrations of taxol used were within the clinically relevant range of achievable plasma drug concentrations (Huizing *et al*, 1993) and were therefore at a level that mitotic spindle-associated microtubules were preferentially targeted rather than the overall cytoskeleton-associated microtubule system (Jordan *et al*, 1993). Nonlimiting doses of taxol permit cells to escape cycle arrest, arising from spindle disruption, providing an opportunity to track apoptotic engagement in potentially therapeutically important subpopulations. Our hypothesis was that although cell death may occur at different stages of progression through mitosis and subsequent cell cycles (Allman and Smith, 2002), the overall kinetics of cell death will relate to the rate of arrival at a ‘critical event window’ in the cell cycle. The results suggest a direct relation between the rate of delivery to a late cell-cycle stage and the subsequent kinetics of cell death. The results also suggest that the net effectiveness of low doses of taxol will be driven primarily by the proliferation kinetics of a tumour cell population despite any differential capacity to rapidly engage cell death pathways as a consequence of genetic status. The findings underline the importance of tracking event resolution over a sufficiently long period to obtain an accurate determination of net effect. Furthermore, our multiparameter analyses of the kinetics of taxol responses in cells with differential capacity to rapidly engage cell death may indicate new rationales for combining taxol with other anticancer agents.

Microtubules are intrinsically dynamic polymers (Wilson and Jordan, 1995) and previous reports have suggested that treatment with high concentrations of taxol induces a cell-cycle arrest in mitosis (Schiff and Horwitz, 1980), which is associated with an increased microtubule polymer mass (Jordan *et al*, 1993). However, treatment of cells with low concentrations of taxol (10 nM) has been suggested to induce a mitotic block by suppressing microtubule dynamics rather than by altering the microtubule polymer mass or inducing bundle formation (Jordan *et al*, 1993). This suggests that the nature of the stress experienced during mitosis, even under conditions of minimal mitotic delay (SU-DHL-4), may be associated with a critical impact of altered microtubule dynamics during mitosis rather than spindle checkpoint activation *per se*.

That p53 plays an important role in the initiation and extent of arrest of cell-cycle progression following DNA damage is now well established (Kastan *et al*, 1991; Kuerbitz *et al*, 1992). The contribution of damage surveillance mechanisms, including wild-type p53, to cell survival following exposure to antitubulin agents is not clear (see the introductory paragraphs), although p53 protein appears to participate in the spindle checkpoint to ensure correct DNA replication before mitosis (Cross *et al*, 1995). p53 has been reported to be a microtubule-associated protein (Maxwell *et al*, 1991) and this raises the possibility that p53 is able to monitor the status of microtubules directly. Aberrant p53 function can suppress apoptosis following DNA damage resulting in a phenotype that is both radiation- and chemoresistant. In contrast, a number of reports have suggested that loss of wild-type p53 function sensitises cells to the action of taxol resulting in both increased G2/M arrest and increased apoptosis.

Formal analysis of p53 involvement in taxol responses will require the analysis of isogenic lines with differential p53 expression and these studies are in progress. Here, we have concentrated on the more specific low-dose effects in a kinetically matched system accessible to high-resolution analysis. Importantly, overall apoptotic kinetics have been appreciated by extended tracking of treated cultures.

These results are in agreement with previous reports that continued DNA replication, in permissive cells, in the presence of mitotic spindle inhibitors results in a drive to polyploidy and abnormal mitotic exit (Jordan *et al*, 1996; Di Leonardo *et al*, 1997). The G1/S competence of DoHH2 may contribute to a postmitotic G1 arrest in agreement with previous studies (Khan and Wahl, 1998; Notterman *et al*, 1998). The development of polyploidy has also been associated with increasing taxol resistance in leukaemia cell lines (Roberts *et al*, 1990). Abnormal mitotic spindle formation prior to the development of polyploidy was confirmed in the present study by confocal microscopy. In both cell lines a similar range of aberrations were observed, with the presence of tripolar spindles being a predominant feature. Confirming studies (data not shown) using a different cell type (Wyllie *et al*, 1999) also revealed a p53-independence of the formation of such aberrant spindles and mitoses for isogenic thyroid-derived K1E7 cells.

The cell lines have similar cell-cycle kinetics, but differential apoptotic responses (Smith *et al*, 1994, 2002). However, a striking feature apparent in the current data is that there is a fixed delay period prior to the onset of apoptosis, which is constant within a given cell line irrespective of taxol dose. Our imaging studies indicate that it is unlikely that the differences in this delay period for the two cell lines relate to differences in the ability of taxol to effect microtubule disruption *per se*. Total exposure time to taxol appears to be less important than whether cells deliver to a critical cell-cycle event during exposure. After this critical event, apoptotic response rate is clearly dose-dependent. It is reasonable to suggest that overt expression of apoptosis merely takes longer in SU-DHL-4 cells. There is a significant correlation (*P*<0.05) between the rate of accumulation of cells in G2/M and the rate of increase of the number of apoptotic cells within the population and this observation is consistent with the hypothesis that the rate at which cells experience mitotic stress determines the subsequent rate of cell death.

We propose a hierarchy of events in the cellular response to taxol (Figure 10). Cells exposed to low concentrations of taxol may undergo a mitotic arrest either by checkpoint activation or by mechanical disruption of microtubules. We also incorporate the possibility that stress-activated protein kinase activity can signal mitotic arrest directly (Wang *et al*, 2000). Cells may subsequently proceed to apoptosis directly (which is rapid) or, in cells that are not subject to a cell-cycle arrest in mitosis, may proceed to undergo a delayed induction of apoptosis that follows aberrant mitotic exit and the formation of polyploid cells.

The mitotic spindle assembly checkpoint has been proposed to regulate taxol-induced apoptosis, with the checkpoint component Bub1 being rapidly phosphorylated following taxol treatment (Taylor *et al*, 2001). Importantly, the taxol concentration used was 1000-fold higher than those used in the present report. Here, we have demonstrated that a prior cell-cycle arrest in mitosis is not required for subsequent progression to apoptosis, that is, cell death can be induced at lower concentrations than those required for cell-cycle arrest. This suggests that taxol can trigger apoptosis without the intervention of spindle checkpoint activation, but still requires cells to experience traverse of the corresponding cell-cycle stage under altered microtubule dynamics.

The potentially wide time course over which apoptotic events may be triggered (upto 48 h) provides few opportunities to correlate biochemical assays with cellular events in heterogenously responding populations monitored here. The antiapoptotic protein survivin has been demonstrated to control microtubule stability and assembly of mitotic spindles and can act to over-ride apoptosis induced by taxol (Li *et al*, 1998; Giodini *et al*, 2002). The relative expression of survivin in different cell systems may therefore also contribute to observed differences in the kinetics of response to taxol. Defining the kinetics of cellular responses to taxol-induced stress in terms of checkpoint activation, cell-cycle arrest, and apoptosis will contribute to a coherent view as to how different agents with different cellular targets may be combined with taxol to enhance therapeutic effectiveness. This is of particular importance where combinations of agents may abrogate taxol activity. We are currently investigating this approach using combinations of ionising radiation and taxol.
